# A new species of *Pristimantis* (Amphibia, Anura, Strabomantidae) from the Pui Pui Protected Forest (central Peru), with comments on *Pristimantis albertus* Duellman & Hedges, 2007

**DOI:** 10.3897/zookeys.994.56277

**Published:** 2020-11-17

**Authors:** Jiří Moravec, Edgar Lehr, Karel Kodejš

**Affiliations:** 1 Department of Zoology, National Museum, Cirkusová 1740, 193 00, Prague 9, Czech Republic National Museum Prague Czech Republic; 2 Department of Biology, Illinois Wesleyan University, P.O. Box 2900, Bloomington, IL 61701, USA Illinois Wesleyan University Bloomington United States of America; 3 Department of Zoology, Faculty of Science, Charles University, Viničná 7, 128 00, Praha 2, Czech Republic Charles University Prague Czech Republic

**Keywords:** Andes, anuran diversity, montane rainforests, *Pristimantis sinschi* sp. nov., *P. sagittulus*, *P. ventrimarmoratus*

## Abstract

We describe a new *Pristimantis* species from the eastern Andes, Región Junín, Peru following an integrative taxonomic approach. The description is based on three adult males (snout-vent length 25.7–28.8 mm) collected in two montane forests between 1615 and 1800 m a.s.l. in the Pui Pui Protected Forest and its close surroundings. The new species is mainly characterised by absence of tympanum, presence of inner tarsal fold, broad horizontal red band across iris, ventre mottled black and cream and ventral surfaces of thighs salmon and grey mottled. Amongst the Amazonian and montane forest *Pristimantis* that have the ventre and groin contrastingly black and cream mottled, *P. sinschi***sp. nov.** is morphologically most similar to *P. lindae* and *P. ventrimarmoratus*. However, DNA barcoding revealed a clear distinction between these three species and placed *P. sinschi***sp. nov.** as sister taxon of *P. lindae*. We designate a lectotype for *P. ventrimarmoratus* and restrict the type locality of this species to “El Topo, R. Pastaza, [Provincia Tungurahua,] E. Ecuador, 4200 feet”. *Pristimantis albertus* and *P. sagittulus* are recorded for the first time in the Región Junín. Additional data on morphology and systematics are provided for *P. albertus*.

## Introduction

**Figure 1.**
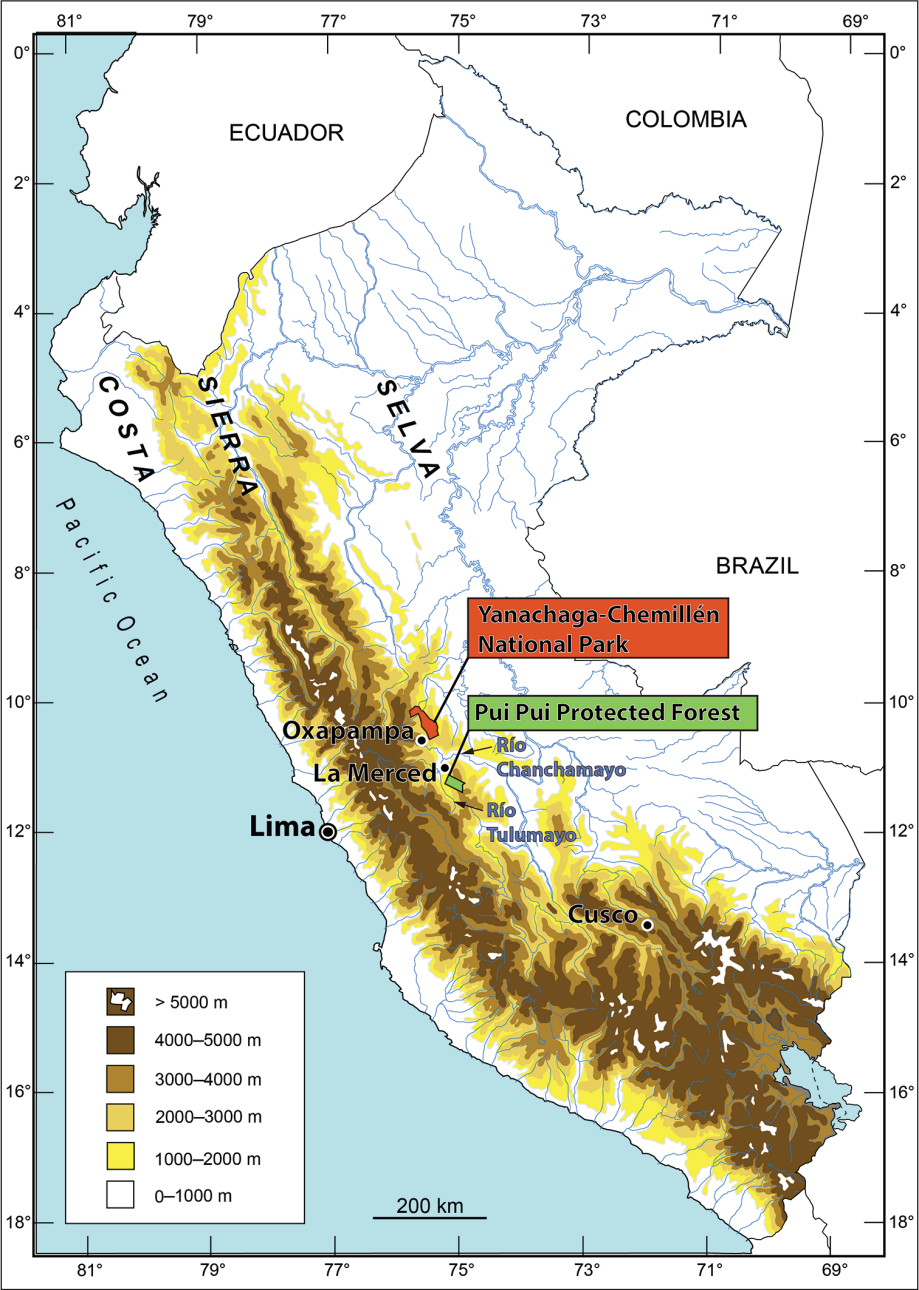
Map of Peru with the Yanachaga-Chemillén National Park (Region Pasco) and the Pui Pui Protected Forest (Region Junín).

Faunal and taxonomic research provides the essential foundation and objectives for the effective conservation of biological diversity. The Pui Pui Protected Forest (PPPF) located in the eastern Andes of Peru (Provincias Chanchamayo, Jauja, Concepción and Satipo, Región Junín; Fig. 1) is a good example of a protected area with a rich, but until recently, practically unexplored fauna and flora. Any proposed inventories, taxonomic studies and conservation assessment of the local fauna and flora are of great importance for proper management of the protected area. Our recent herpetological surveys (first in the area) revealed that the amphibian and reptile fauna of the PPPF is remarkably diverse with a high degree of local endemism. We obtained new faunal records extending the known ranges of several species (e.g. *Euspondylus excelsum* Chávez, Catenazzi & Venegas, 2017: Lehr et al. 2018) and discovered nine taxa new to science (six anuran and one reptile species, two reptile genera; for example, Lehr and Moravec 2017; Lehr and von May 2017; Lehr et al. 2017b, c; Lehr et al. 2018; Moravec et al. 2018; Lehr et al. 2020). Nevertheless, it appears that our knowledge of the real species richness of the local amphibian and reptile fauna is still incomplete. Thorough genetic analyses of our collected *Pristimantis*, specimens from the PPPF revealed unrecognised taxa and new records for known taxa.

Herein, we describe a new species of *Pristimantis* Jiménez de la Espada, 1870 which phenotypically resembles *P. diadematus* (Jiménez de la Espada, 1875); *P. divnae* Lehr and von May, 2009; *P. eurydactylus* (Hedges & Schlüter, 1992); *P. lindae* (Duellman, 1978); *P. orcus* Lehr, Catenazzi & Rodríguez, 2009; and *P. ventrimarmoratus* (Boulenger, 1912). Furthermore, we provide the first records for *Pristimantis albertus* Duellman & Hedges, 2007 and *P. sagittulus* (Lehr, Duellman & Aguilar, 2004) for the Región Junín and additional data on systematics and morphology for *P. albertus*.

## Material and methods

### Morphological characters

The format for the description follows Lynch and Duellman (1997), except that the term dentigerous processes of vomers is used instead of vomerine odontophores (Duellman et al. 2006) and diagnostic characters are those of Duellman and Lehr (2009). Taxonomic classification follows Hedges et al. (2008), except that we followed Pyron and Wiens (2011) for family placement and Padial et al. (2014) for names of *Pristimantis* species groups. The holotype was fixed in 96% ethanol and stored in 70% ethanol. Liver tissue of the holotype was taken for genetic analyses. Sex and maturity of specimens were identified by observing secondary sexual characters (vocal slits) and gonads through dissections. Specimens were considered juveniles when gonads were too small to distinguish between sexes. We used maximum known SVL for males within a species to recognise smallest body size as recommended by Lehr and Coloma (2009). We measured the following variables to the nearest 0.1 mm with digital calipers under a stereomicroscope: snout-vent length (SVL, straight length distance from tip of snout to vent) , tibia length (TL, distance from the knee to the distal end of the tibia), foot length (FL, distance from proximal margin of inner metatarsal tubercle to tip of Toe IV), head length (HL, from angle of jaw to tip of snout), head width (HW, at level of angle of jaw), horizontal eye diameter (ED), interorbital distance (IOD), upper eyelid width (EW), internarial distance (IND), eye-nostril distance (E-N, straight line distance between anterior corner of orbit and posterior margin of external nares) and horizontal tympanum diameter (TD). Fingers and toes are numbered preaxially to postaxially from I–IV and I–V, respectively. We compared the lengths of toes III and V by adpressing both toes against Toe IV; lengths of fingers I and II were compared by adpressing the fingers against each other. Drawings were made by EL using a stereomicroscope and a camera lucida. Photographs taken by JM and EL were used for descriptions of colouration in life.

Comparisons of congeners focused on phenotypically-similar species from Ecuador and Peru and those with close phylogenetic relationships as recovered in our trees. Information on species for comparative diagnoses was obtained from Duellman and Lehr (2009) and original species descriptions. For specimens examined, see paragraph Comparative specimens examined. Codes of collections are: QCAZ = Museo de Zoología of the Pontificia Universidad Católica del Ecuador; MUSM = Museo de Historia Natural Universidad Nacional Mayor de San Marcos, Lima, Peru; NMP-P6V = National Museum Prague, Czech Republic. Threat status was assessed using the IUCN criteria (2016).

### Molecular analysis

#### Taxon sampling

**Figure 2.**
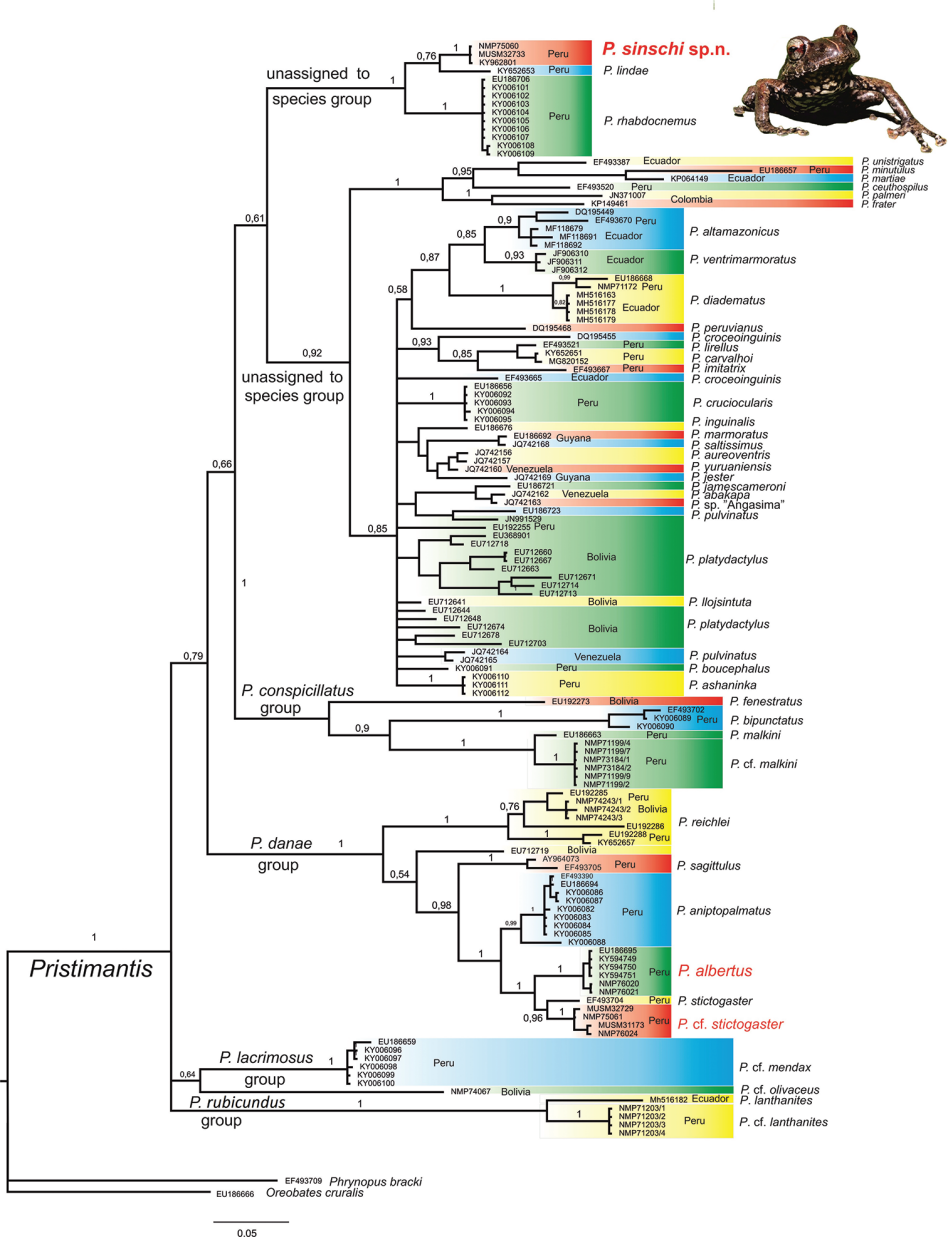
The Bayesian phylogenetic tree of selected South American *Pristimantis*; for taxon sampling design, see Material and Methods. Nodes with less than 50% of the post burn-in tree samples were collapsed. Posterior probability values (pp) given only for main lineages. The new species, *P. sinschi* sp. nov., is a member of a monophyletic clade, yet unnamed species Group, comprising species from Peruvian Cordillera Oriental – *P. lindae* and *P. rhabdocnemus*. The PPPF representatives of *P. danae* species Group form two distinct genetic lineages, one conspecific with *P. albertus* and the second closer to *P. stictogaster* (labelled as P. cf. stictogaster). Species-group names follow Padial et al. (2014). DNA sequences (for 12S rRNA and 16S rRNA) of taxa retrieved from GenBank are labelled by standard GenBank accession numbers (in cases where both 16S rRNA and 12S rRNA for the same specimen are under different accession numbers, taxon is labelled with 16S rRNA number), all other codes stand for our new material.

Proceeding from our previous study (Lehr et al. 2017a), we included samples of various *Pristimantis* species collected by us in the Pui Pui Protected Forest (PPPF) during the surveys between 2012 and 2014 and from the area of nearby montane regions of the Cordillera Yanachaga. A list of the new genetically-investigated material and their GenBank accession numbers are presented in Table 1. For the final dataset, we retrieved additional sequences conspecific or presumably related with our samples from GenBank to show phylogenetic positions of our new material in relation to DNA sequences published earlier (most importantly in the review by Hedges et al. 2008). We also retrieved species known to occur in the Cordillera Yanachaga Region (Duellman and Hedges 2005, 2007) including holotypes of *P. albertus* Duellman & Hedges, 2007 and *P. stictogaster* Duellman & Hedges, 2005. For comparison of the new species with morphologically-similar *P. lindae* and *P. ventrimarmoratus*, we used DNA sequences obtained by Arteaga-Navarro et al. (2011) and von May et al. (2017). As outgroups, we used the strabomantid genera *Oreobates* Jiménez de la Espada, 1872 (*O. cruralis* (Boulenger, 1902)) and *Phrynopus* Peters, 1873 (*P. bracki* Hedges, 1990), retrieved from GenBank. The final dataset was composed of 134 samples of 49 nominal taxa, including the new species and outgroups. All sequences acquired from GenBank can be identified by the GenBank accession numbers as given in Fig. 2.

**Table 1.** Names of taxa, museum numbers, field data and GenBank accession numbers of newly-genetically investigated material. For other abbreviations, see text. PPPF = Pui Pui Protected Forest.

Species	Museum number	Locality	Coordinates	Elevation (m)	Collectors; year	GenBank Accession number	
						16S	12S
*P. sinschi* sp. nov. (holotype)	MUSM 32733	PPPF, Peru	11°12'38.5"S, 74°57'28.9"W	1800	E. Lehr, J. Moravec; 2014	MW075408	MW075426
*P. sinschi* sp. nov.	NMP-P6V 75060	PPPF, Peru	11°12'38.5"S, 74°57'28.9"W	1615	E. Lehr, J. Moravec; 2014	MW075407	MW075425
*P. albertus*	NMP-P6V 76020	PPPF, Peru	11°07'37.2"S, 75°10'37.0"W	1970	E. Lehr, J. Moravec, J.C. Cusi, R. von May; 2013	MW075393	MW075414
*P. albertus*	NMP-P6V 76021	PPPF, Peru	11°07'37.2"S, 75°10'37.0"W	1970	E. Lehr, J. Moravec, J.C. Cusi, R. von May; 2013	MW075394	MW075415
P. cf. stictogaster	MUSM 32729	PPPF, Peru	11°12'38.5"S, 74°57'28.9"W	1700	E. Lehr, J. Moravec; 2014	MW075395	MW075417
P. cf. stictogaster	NMP-P6V 75061	PPPF, Peru	11°12'38.5"S, 74°57'28.9"W	1750	E. Lehr, J. Moravec; 2014	MW075396	MW075419
P. cf. stictogaster	MUSM 31173	PPPF, Peru	11°15'16.1"S, 74°53'30.7"W	1615	E. Lehr, R. von May; 2012	–	MW075416
P. cf. stictogaster	NMP-P6V 76024	PPPF, Peru	11°15'16.1"S, 74°53'30.7"W	1615	E. Lehr, R. von May; 2012	–	MW075418
P. cf. malkini	NMP-P6V 71199/2	Tarapoto, Peru	03°48'17"S, 73°24'19"W	95	J. Moravec; 2001	MW075401	MW075423
P. cf. malkini	NMP-P6V 71199/4	Tarapoto, Peru	03°48'17"S, 73°24'19"W	95	J. Moravec; 2001	MW075399	–
P. cf. malkini	NMP-P6V 71199/7	Tarapoto, Peru	03°48'17"S, 73°24'19"W	95	J. Moravec; 2001	MW075397	–
P. cf. malkini	NMP-P6V 71199/9	Tarapoto, Peru	03°48'17"S, 73°24'19"W	95	J. Moravec; 2001	MW075402	–
P. cf. malkini	NMP-P6V 73184/1	Tarapoto, Peru	03°48'17"S, 73°24'19"W	95	J. Moravec; 2002	MW075400	MW075424
P. cf. malkini	NMP-P6V 73184/2	Tarapoto, Peru	03°48'17"S, 73°24'19"W	95	J. Moravec; 2002	MW075398	–
*P. diadematus*	NMP-P6V 71172	Tarapoto, Peru	03°48'17"S, 73°24'19"W	95	J. Moravec; 2001	MW075409	MW075420
P. cf. lanthanites	NMP-P6V 71203/1	Tarapoto, Peru	03°48'17"S, 73°24'19"W	95	J. Moravec; 2001	MW075405	MW075421
P. cf. lanthanites	NMP-P6V 71203/2	Tarapoto, Peru	03°48'17"S, 73°24'19"W	95	J. Moravec; 2001	MW075403	–
P. cf. lanthanites	NMP-P6V 71203/3	Tarapoto, Peru	03°48'17"S, 73°24'19"W	95	J. Moravec; 2001	MW075404	–
P. cf. lanthanites	NMP-P6V 71203/4	Tarapoto, Peru	03°48'17"S, 73°24'19"W	95	J. Moravec; 2001	MW075406	–
P. cf. olivaceus	NMP-P6V 74067	Palmira, Bolivia	10°35'S, 65°44'W	150	J. Moravec; 2007	MW075410	MW075422
*P. reichlei*	NMP-P6V 74243/1	San Antonio, Bolivia	11°29'S, 68°52'W	250	J. Moravec; 2007	MW075411	MW075427
*P. reichlei*	NMP-P6V 74243/2	San Antonio, Bolivia	11°29'S, 68°52'W	250	J. Moravec; 2007	MW075413	–
*P. reichlei*	NMP-P6V 74243/3	San Antonio, Bolivia	11°29'S, 68°52'W	250	J. Moravec; 2007	MW075412	–

### DNA extraction, PCR, sequencing and sequence alignment

Genomic DNA was extracted from tissues stored in 96% ethanol. A fragment of the mitochondrial gene for 16S rRNA (16S), which is commonly used in the amphibian DNA barcoding (Vences et al. 2012), was targeted using the primers 16SL1 and 16SH1 adapted or directly taken from Palumbi et al. (1991). For primer sequences and PCR conditions, see Moravec et al. (2009).

In the case of *Pristimantis ventrimarmoratus*, only the 12S sequence was available in the GenBank database. Therefore, for the purpose of comparison, a fragment of 12S rRNA (*12S*) was sequenced for the holotype of the new species and 13 other selected specimens (Table 1; at least one specimen per species). The 12S fragment was amplified using primers 12Sa (5’-AAACTGGGATTAGATACCCCACTAT – 3’) and 12Sb (5’-GAGGGTGACGGGCGGTGTGT-3’) and 12Sb (5’-GAGGGTGACGGGCGGTGTGT-3’), taken from Kocher et al. (1989), using the PCR protocol described in Moravec et al. (2009), with an annealing temperature of 48 °C. New sequences have been deposited in GenBank (MW075393–MW075427). The multiple sequence alignment was performed in Geneious 11.1.5 (Kearse et al. 2012), using implemented MUSCLE algorithm (Edgar 2004), producing a 710 bp-long concatenated alignment (incomplete matrix, 464 bp 16S and 246 bp 12S). Ambiguously-aligned positions were eliminated by Gblocks v0.91b under options for a less stringent selection (Castresana 2000).

### Phylogenetic analysis

The Bayesian Inference (BI) was applied to construct a phylogenetic tree. First, the software PartitionFinder 2.1.1, using the PhyML algorithm (Lanfear et al. 2012), was used to find the best-fitting model for nucleotide evolution, which was the GTR+I+G model, based on both the Akaike and Bayesian information criteria. The same model was rated as best-fitting for both 16S rRNA and 12S rRNA under partitioned search setting. The BI analysis was run in MrBayes 3.2.7a (Ronquist et al. 2012) with two runs and four chains in each run for 6 × 10^6^ generations, sampling every 100^th^ generation. Appropriate sampling was controlled by examining the stationarity of log-likelihood scores against the generation time using Tracer v1.6 (Rambaut et al. 2013; all parameters had an effective sample size > 1000) and convergence between the two simultaneous runs was confirmed by the convergence diagnostics of the average standard deviation of split frequencies and the potential scale reduction factor values. From the sampled trees, 25% were discarded as burn-in and a 50% majority-rule consensus tree was produced from the remaining post-burn-in trees. The posterior probabilities (pp) were calculated as the frequency of samples recovering any particular clade (Huelsenbeck and Rannala 2004). Genetic uncorrected *p*-distances were calculated in PAUP* (Swofford 2003).

## Results

### Molecular phylogenetic analyses

The obtained phylogenetic tree (Fig. 2) shows high support for six distinct evolutionary lineages within *Pristimantis* which are consistent with clades uncovered by Padial et al. (2014) and reconstructed also in our previous study (Lehr et al. 2017a). The newly-recognised species, *Pristimantis sinschi* sp. nov., belongs to a highly-supported clade (unassigned to any species group according to Padial et al. 2014) with *P. rhabdocnemus* and *P. lindae* as closest relatives (*P. lindae* in sister position). However, the average genetic uncorrected *p*-distance of 4.02% in the 16S barcode supports their status as separate species (Table 2).

**Table 2.** Mean uncorrected genetic *p*-distance values of 16S rRNA barcode for species from the clade containing *Pristimantis sinschi* sp. nov. and for *P. albertus*–*P. stictogaster* clade within *P. danae* species Group.

	Species	1	2	3	4	5	6	7
1	*P. sinschi* sp. nov.	0.0000						
2	*P. lindae*	0.0402	0.0000					
3	*P. rhabdocnemus*	0.0535	0.0713	0.0136				
4	*P. albertus* GeneBank	0.1721	0.1691	0.1808	0.0000			
5	*P. albertus* this study	0.1721	0.1692	0.1809	0.0067	0.0000		
6	*P. stictogaster*	0.1611	0.1514	0.1720	0.0468	0.0497	0.0000	
7	P. cf. stictogaster	0.1585	0.1571	0.1745	0.0444	0.0472	0.0283	0.0023

Morphologically-similar species *P. ventrimarmoratus* (individuals from Ecuador, GenBank accession numbers: JF906310.1, JF906311.1 and JF906312.1) clusters within the species-rich clade comprising species from the Andes and the Guiana Shield, as well as lowland species from the intervening Amazon, with *P. altamazonicus* (Barbour & Dunn, 1921) and *P. brevicrus* (Andersson, 1945) as closest relatives (recently revalidated *P. brevicrus*, see Ortega-Andrade et al. 2017, is not in the tree, its position being verified separately). Average genetic uncorrected *p*-distance for 12S sequences between *P. ventrimarmoratus* and *P. sinschi* sp. nov. is over 10%. However, the situation concerning *P. ventrimarmoratus* is more complex and the exact identity of this species needs to be clarified. There are four syntypes of *Hylodes ventrimarmoratus* Boulenger, 1912: BMNH 1947.2.15.73 (formerly 1911.12.12.77) from “Chanchamayo, [Departamento Junín,] Peru, 2600 feet” and 1947.2.15.74–76 (formerly 1911.1.1.51–53) from “El Topo, R. Pastaza, [Provincia Tungurahua,] E. Ecuador, 4200 feet” (Duellman and Lehr 2009, Frost 2020). The Ecuadorian syntypes apparently belong to the sequenced Ecuadorian population of *Pristimantis ventrimarmoratus* because their locality lies between the collecting sites of the sequenced specimens (ca. 158 km SW of the locality of QCAR 41938 and QCAZ 42048 and ca. 176 km N of the locality of QCAZ 36403; see Arteaga-Navarro and Guayasamin 2011). Therefore, the Ecuadorian syntypes of *Hylodes ventrimarmoratus* can be regarded as the type specimens of *Pristimantis ventrimarmoratus* (Boulenger, 1912). Contrary to this, the Peruvian syntype of *Hylodes ventrimarmoratus* may be conspecific with *Pristimantis sinschi* sp. nov. It also originates from the Provincia Chanchamayo and displays unusual similarity to the new species (for comparison, see the photographs in Frenkel et al. 2018). To solve this taxonomic issue, we designate the syntype BMNH 1947.2.15.74 as the lectotype of *Hylodes ventrimarmoratus*. In consequence, the type locality of this taxon is “El Topo, R. Pastaza, [Provincia Tungurahua,] E. Ecuador, 4200 feet” (see the part Taxonomy).

The PPPF*Pristimantis* individuals, morphologically resembling *P. albertus*, belong to two separate lineages within the *P. danae* species Group. Specimens NMP-6V 76020-21 from the area of Rio Huatziroki are genetically very close to the holotype of *P. albertus* (KU 291675, GenBank accession number: EU186695; Duellman and Hedges 2007 – average uncorrected genetic *p*-distance = 0.6 %). Specimens MUSM 31173, 32729 and NMP-6V 75061, 76024 collected in the area of Río Bravo and surroundings of Nueva Florida (for coordinates, see Table 1) cluster more closely with *P. stictogaster* and we tentatively name them P. cf. stictogaster. The average genetic uncorrected *p*-distance between P. cf. stictogaster and holotype of *P. stictogaster* is 2.8% (Fig. 2, Table 2).

### Taxonomy

#### Pristimantis ventrimarmoratus

Taxon classificationAnimaliaAnuraStrabomantidae

(Boulenger, 1912)

DC9CB5B5-A377-5F7A-B381-A4933B93A23B

Hylodes ventrimarmoratus Boulenger, 1912

##### Designated lectotype

**(hoc loco)**: BMNH 1947.2.15.74

##### Paralectotypes.

BMNH 1947.2.15.75 and BMNH 1947.2.15.76

##### Type locality.

“El Topo, R. Pastaza, [Provincia Tungurahua,] E. Ecuador, 4200 feet”

##### Note.

The fourth original syntype BMNH 1947.2.15.73 may be conspecific with *Pristimantis sinschi* sp. nov. but its accurate determination remains open.

##### Synonymy.

*Eleutherodactylus ventrivittatus* Andersson, 1945; Type locality: “Ambitagua [= Abitagua], Rio Pastaza”, Provincia Tungurahua or Pastaza, Ecuador (according to Frost 2020).

#### Pristimantis sinschi

Taxon classificationAnimaliaAnuraStrabomantidae

Lehr, Moravec & Kodejš sp. nov.

5EE47F1E-BB19-56CC-890E-DBDF3E835294

http://zoobank.org/3E920AC3-5123-4B26-8B7A-84BBF3F5B68E

Figs 4 , 5 , 6 , Tables 1 , 2 , 3 Suggested English name: Ulrich Sinsch’s Rubber Frog Suggested Spanish name: Rana cutín de Sinsch

Pristimantis sp. A – Lehr et al. (2017a)
Pristimantis sp. nov. – Lehr and Moravec (2017)

##### Holotype.

**Figure 3.**
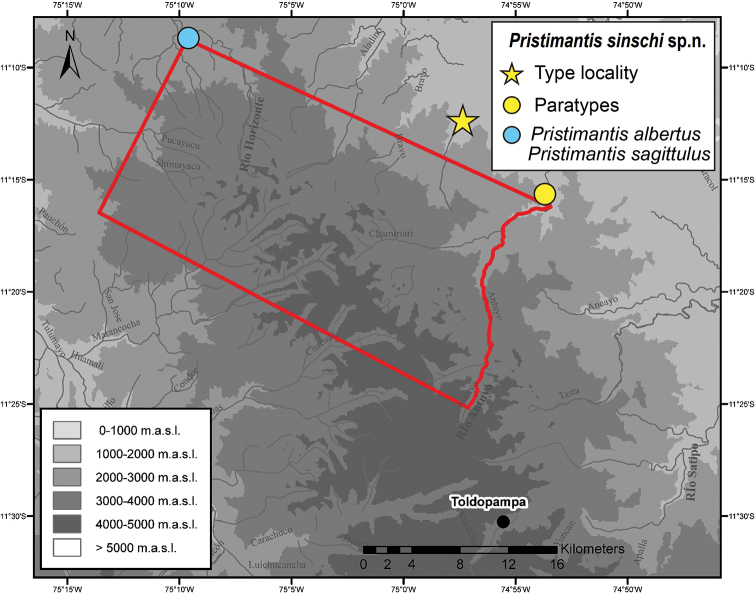
Pui Pui Protected Forest (red frame) with collecting sites of *Pristimantis sinschi* sp. nov. (yellow symbols) and *P. albertus* and *P. sagittulus* (blue symbol). Modified after Lehr et al. (2017).

MUSM 32733 (field number IWU 367), GenBank accession numbers MW075408 (16S rRNA) and MW075426 (12S rRNA), an adult male from the Pui Pui Protected Forest (11°12'38.5"S, 74°57'28.9"W; Figs 1, 3), 1800 m a.s.l., Distrito Pichanaqui, Provincia Chanchamayo, Región Junín, Peru, collected on 16 May 2014 by Edgar Lehr and Jiří Moravec.

##### Paratypes.

Two adult males: NMP-P6V 75060 (field number IWU 359), GenBank accession numbers MW075407 (16S rRNA) and MW075425 (12S rRNA), collected at the type locality on 14 May 2014 by Edgar Lehr and Jiří Moravec. MUSM 31165 (field number IWU 123), GenBank accession number KY962801, collected at the entrance of the Pui Pui Protected Forest (11°15'16.1"S, 74°53'30.7"W; Figs 1, 3), 1615 m a.s.l., reached from Nueva Florida in ca. 8 hours walk on 1 May 2012 by Edgar Lehr, and Rudolf von May.

##### Generic placement.

We assign this species to *Pristimantis*, based on our molecular data (Fig. 2) and general morphological similarity to other members of the genus.

##### Diagnosis.

A new species of *Pristimantis* not assigned to any species group having the following combination of characters: (1) Skin on dorsum shagreen with many small subconical tubercles and a narrow, hairline mid-dorsal fold, skin on ventre areolate; discoidal and thoracic folds present; dorsolateral folds absent; (2) tympanic membrane and tympanic annulus absent; (3) snout short, rounded in dorsal and lateral views; (4) upper eyelid without enlarged tubercles; EW slightly shorter than IOD; cranial crests absent; (5) dentigerous processes of vomers present; (6) males without vocal slits; nuptial pads absent; (7) Finger I shorter than Finger II; discs of digits broadly expanded, elliptical; (8) fingers with lateral fringes; (9) small conical ulnar and tarsal tubercles present; (10) heel without conical tubercles; short inner tarsal fold present; (11) inner metatarsal tubercle ovoid, 3 times as large as outer; outer metatarsal tubercle small, ovoid; low, numerous supernumerary plantar tubercles; (12) toes with lateral fringes; basal toe webbing absent; Toe V longer than Toe III; toe discs slightly smaller than those on fingers; (13) in life, dorsal ground colouration greenish-grey, reddish-brown or brown with or without a hairline mid-dorsal tan stripe; canthal stripe absent, supratympanic stripe greyish-brown; groin black with cream blotches, anterior surfaces of thighs and ventral surface of shanks black; ventral surfaces of thighs salmon and grey mottled; ventre black and cream mottled; iris pale bronze with fine black vermiculation and broad median red band through pupil and a narrow black vertical streak from pupil across lower half of iris; (14) SVL in adult males 25.7–28.8 mm (n = 3), females unknown.

##### Comparison.

Phylogenetically, *Pristimantis sinschi* and *P. lindae* from southern Peru (Región Cusco) are sister taxa. Both species have the dorsum shagreen with subconical tubercles, ventre areolate, dorsolateral folds absent, dentigerous processes of vomers, finger and toe discs expanded, fingers and toes with lateral fringes, tarsal folds, males with vocal slits and groin and ventre cream with black reticulations. However (characters for *P. sinschi* in parenthesis), *P. lindae* has a tympanum (absent), the single known male MUSM 26528 has nuptial pads (absent), ventral surfaces of thighs pale brown and grey mottled (salmon and grey mottled) and the iris with a median horizontal dark reddish-brown streak (red) (Duellman 1978).

*Pristimantis sinschi* is morphologically similar to five other *Pristimantis* (*P. diadematus*, *P. divnae*, *P. eurydactylus*, *P. orcus* and *P. ventrimarmoratus*) from the Amazonian lowlands and lower montane forests which have the ventre and groin contrastingly patterned in black and cream and an inner tarsal fold. However, *P. sinschi* is readily distinguished from its congeners (except for *P. ventrimarmoratus*) by lacking a tympanum and having the ventral surfaces of thighs orange brown and grey mottled. Characters for *P. sinschi* are in parenthesis in the following. Furthermore, *P. diadematus* has the ventral skin smooth (areolate), males with vocal slits (absent) and nuptial pads (absent) and the iris greenish-bronze with a median horizontal red streak or reddish-copper (pale bronze with fine black vermiculation and broad median horizontal red band) (Duellman and Lehr 2009). Male *P. divnae* have a much smaller SVL of 22.8–23.4 mm, n = 2 (25.7–28.8 mm, n = 3), scapular region with a W-shaped ridge (hour-glass shaped ridge) and iris golden with black reticulations and fine narrow black bars forming a cross or T (pale bronze with fine black vermiculation and broad median horizontal red band and a vertical streak at its lower half) (Lehr and von May 2009). Male *P. eurydactylus* lack vocal slits, have nuptial pads (absent), a maximum known SVL of 31.8 mm (28.8 mm, and scapular region with a W-shaped ridge (hour-glass shaped ridge) (Hedges and Schlüter 1992). Male *P. orcus* have nuptial pads present (absent), have a much smaller SVL of 20.0–25.1, n = 4 (25.7–28.8 mm, n = 3), the groin white or whitish-blue and black (groin black with cream blotches) and the iris gold with a copper tint and fine black reticulations (pale bronze with fine black vermiculation and broad median horizontal red band) (Lehr et al. 2009). *Pristimantis ventrimarmoratus* and *P. sinschi* both lack a tympanum and males are without vocal slits. However, male *P. ventrimarmoratus* have nuptial pads (absent), have a smaller SVL 17.8–25.5 mm, n = 8 (25.7–28.8 mm, n = 3) and the iris pale bronze with fine black flecks (pale bronze with fine black vermiculation and broad median horizontal red band) (Lynch and Duellman 1980).

##### Holotype description.

**Figure 4.**
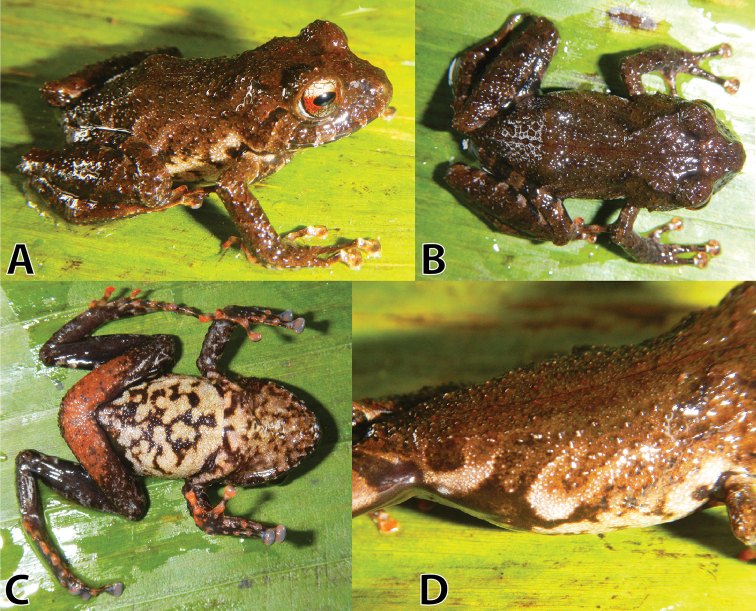
Life holotype (MUSM 32733, adult male, SVL 25.7mm) of *Pristimantis sinschi* sp. nov. in dorsal (**A**), dorsolateral (**B**), ventral (**C**) and lateral (**D**) views. Photos by E. Lehr.

Head slightly narrower than body, slightly wider than long; head length 42% of SVL; head width 44% of SVL; cranial crests absent; snout moderately long, rounded in dorsal and lateral views (Fig. 4A, B); eye-nostril distance 72% of eye diameter; nostrils slightly protuberant, directed dorsolaterally; canthus rostralis moderately long, broadly rounded in lateral view, weakly concave in dorsal view; loreal region weakly concave; lips rounded; upper eyelid each with several small subconical tubercles; upper eyelid width 86% of IOD; occipital and scapular region with several enlarged conical tubercles and with an hourglass-shaped fold from posterior margin of upper eyelid slightly passing the level of arm insertion; supratympanic fold short and narrow, extending from posterior margin of upper eyelid slightly curved to level of mouth corner; tympanic membrane and annulus absent; one conical postrictal tubercle present bilaterally. Choanae small, ovoid, not concealed by palatal shelf of maxilla; dentigerous processes of vomers oblique, moderately-sized, widely separated, each bearing four teeth; tongue discoidal, not notched posteriorly, covering entire floor of mouth, posterior fifth and lateral parts free.

Skin on dorsum and flanks shagreen with many small conical tubercles, dorsolateral folds absent, a narrow, hairline mid-dorsal fold present from snout towards cloacal sheath; skin on throat, chest and belly areolate; discoidal and thoracic folds present, weakly defined; cloacal sheath short.

**Figure 5.**
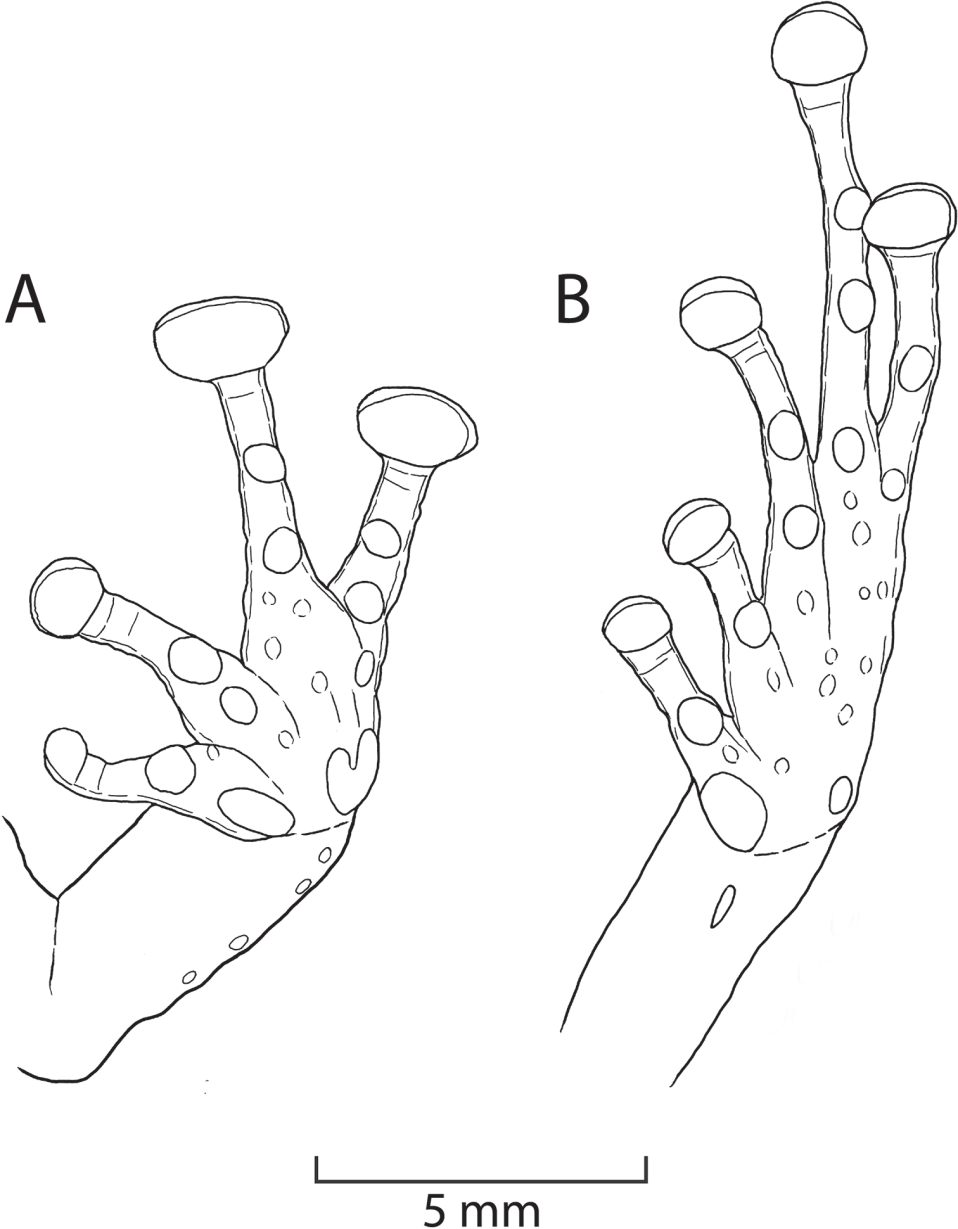
Ventral views of hand (**A**) and foot (**B**) of holotype of *Pristimantis sinschi* sp. nov. Drawings by E. Lehr.

Outer ulnar surface with four (left side) and three (right side) minute low tubercles; palmar tubercle partially divided distally; thenar tubercle ovoid; subarticular tubercles well defined, round in ventral view, conical in lateral view; supernumerary tubercles distinct, ovoid, subconical, approximately half the size of subarticular tubercles; fingers with narrow lateral fringes, much broader at base of fingers; Finger I shorter than Finger II; discs on digits of fingers broadly expanded (about 1.5 times width of digit proximal to disc), elliptical (Fig. 5A).

Hind limbs long, slender, tibia length 54% of SVL; foot length 51% of SVL; upper surfaces of hind limbs shagreen with many subconical tubercles; inner surface of thighs smooth, posterior and ventral surfaces of thighs areolate; heels without enlarged conical tubercles; outer surface of tarsus with scattered minute low tubercles; inner tarsal fold present, short and narrow, most distinct at its anterior third; inner metatarsal tubercle prominent, ovoid, three times the size of ovoid outer metatarsal tubercle; subarticular tubercles well defined, round in ventral view, conical in lateral view; plantar supernumerary tubercles distinct, about half the size of subarticular tubercles; toes with narrow lateral fringes; basal webbing absent; discs broadly expanded, elliptical, less expanded than those on fingers; relative length of toes: 1 < 2 < 3 < 5 < 4; disc on Toe III reaching distal subarticular tubercle on Toe IV, disc on Toe V extends distal subarticular tubercle on Toe IV (Fig. 5B).

In life (Fig. 4), dorsum brown with narrow reddish-brown mid-dorsal stripe and weakly-defined reddish-brown interorbital bar; scapular region with widely-separated narrow dark greyish-brown X-shape (Fig. 4B); upper lip with two greyish-brown subocular bars and a greyish-brown supratympanic bar (Fig. 4A); arms and hind legs brown with diagonal black bars, finger and toe discs pale salmon; lower half of flanks cream, upper half of flanks with irregular-shaped dark brown diagonal stripes (Fig. 4A, D), groin and axilla black with cream blotches, posterior surface of thighs black, anterior surface of thighs with diagonal black bars interspaced with pale brown bars; throat greyish-cream with black mottling, chest and belly cream and black mottled, ventral surfaces of thighs salmon and grey mottled, arms and hind legs black and pale grey mottled, with hand and feet surfaces pale grey with palmar and plantar tubercles salmon and discs of fingers I–II and discs of toes I–III salmon, other discs pale grey (Fig. 4C); iris pale bronze with fine black vermiculation and broad median red band through pupil and a narrow black vertical streak from pupil across lower half of iris (Fig. 4A).

In alcohol, general colouration pattern is as described for the holotype in life, except for brown which is pale brown, black which is dark brown, salmon which is cream and pale grey which is brown. Iris is pale grey.

Holotype measurements (in mm): SVL 25.7; TL 13.9; FL 13.0, HL 10.7; HW 11.2; ED 3.9; IOD 3.5; EW 3.0; IND 2.3; E-N 2.8.

##### Variation.

**Figure 6.**
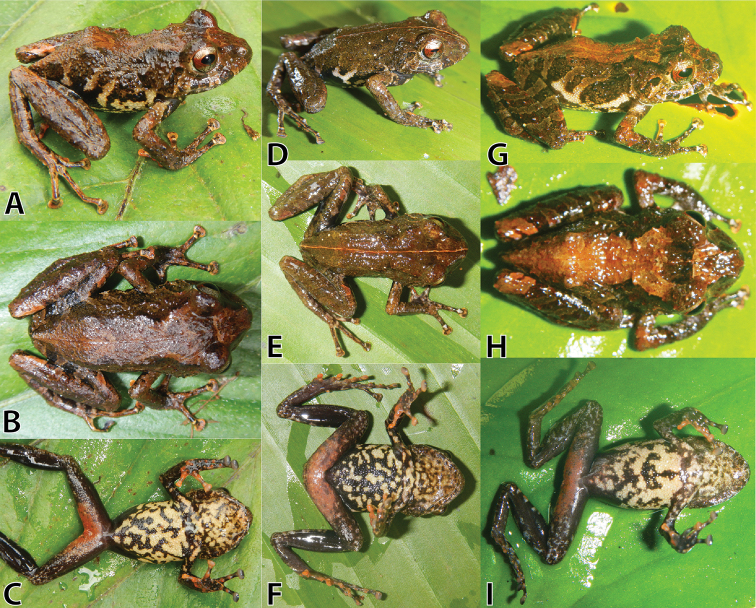
Variation of life male paratypes (**A–F**) and one uncollected specimen (**G–I**) of *Pristimantis sinschi* sp. nov. in dorsolateral, dorsal and ventral views **A–C** (MUSM 31165, SVL 25.8 mm) **D–F** (NMP-P6V 75060, SVL 28.8 mm) and **G–I** (uncollected). Photos by E. Lehr.

All paratypes are similar to the holotype regarding morphology (see Table 3) and colouration pattern (Fig. 6). One specimen has a narrow tan mid-dorsal stripe from snout to cloaca (NMP-P6V 75060, Fig. 6D, E) and all specimens have short and narrow ridges on the anterior flanks (Fig. 6A, B, D, G). One uncollected specimen has a brownish-orange dorsal band (Fig. 6H) and the occipital region with prominent conical tubercles (Fig. 6G).

**Table 3.** Measurements (in mm) of male type specimens of *Pristimantis sinschi* sp. nov. For abbreviations, see Material and methods.

Character	MUSM 32733	MUSM 31165	NMP-P6V 75060	Ranges followed by means and SD in parenthesis
SVL	25.7	25.8	28.8	25.7–28.8 (26.8 ± 1.8)
TL	13.9	14.2	15.6	13.9–15.6 (14.6 ± 0.9)
FL	13.0	13.0	14.6	13.0–14.6 (13.5 ± 0.9)
HL	10.7	10.7	11.5	10.7–11.5 (11.0 ± 0.5)
HW	11.2	11.4	11.8	11.2–11.8 (11.5 ± 0.3)
ED	3.9	3.5	3.8	3.5–3.9 (3.7 ± 0.2)
IOD	3.5	3.0	3.2	3.0–3.5 (3.2 ± 0.3)
EW	3.0	2.4	2.8	2.4–3.0 (3.1 ± 0.7)
IND	2.3	2.3	2.4	2.3–2.4 (2.3 ± 0.1)
N–E	2.8	2.6	2.8	2.6–2.8 (2.7 ± 0.1)

##### Distribution and natural history.

**Figure 7.**
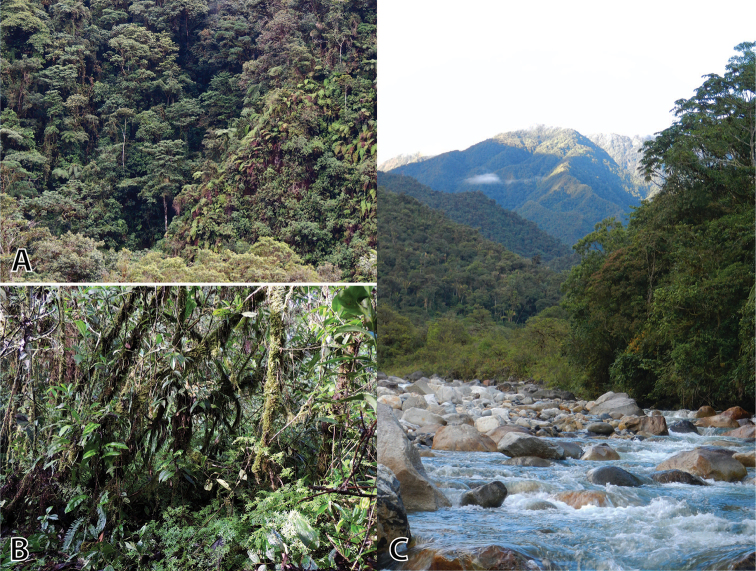
Collecting sites in montane forests of *Pristimantis sinschi* sp. nov. Type locality (**A, B**) in the close surrounding of the Pui Pui Protected Forest at 1800 m a.s.l. Second known locality (**C**) at the entrance of the Pui Pui Protected Forest at 1615 m a.s.l. Photos by J. Moravec (**A, B**) and E. Lehr (**C**).

*Pristimantis sinschi* is known from two localities in montane forest of the Pui Pui Protected Forest and its close surroundings in the eastern Andes between 1615 and 1800 m a.s.l. in the Región Junín (Figs 3, 7). The type locality is a primary montane forest with dense vegetation including ferns, tree ferns and epiphytes (bromeliads, mosses). All three frogs were found at night on the vegetation between 80 and 250 cm above ground. The holotype was found on a leaf of a tree fern at 250 cm aboveground, NMP-P6V 75060 was found on a leaf at 150 cm aboveground and MUSM 31165 was found on a leaf at 80 cm aboveground. In the Pui Pui Protected Forest, *Pristimantis sinschi* occurs syntopically with *P. albertus*; *P. ashaninka* Lehr & Moravec, 2017; *P. aniptopalmatus* (Duellman & Hedges, 2005); *P. bipunctatus* (Duellman & Hedges, 2005); *P. cruciocularis* (Lehr, Lundberg, Aguilar & von May, 2006), P. cf. platydactylus (Boulenger, 1903), *P. sagittulus* and P. cf. stictogaster. According to the sparse data available, we here classify *P. sinschi* as “Data Deficient” according to the IUCN Red List criteria.

##### Etymology.

We dedicate this species to our colleague and friend Prof. Dr. Ulrich Sinsch in recognition of his important contributions to the South American and African herpetology. The specific epithet is used as a noun in apposition.

#### Pristimantis albertus

Taxon classificationAnimaliaAnuraStrabomantidae

Duellman & Hedges, 2007

91B6642F-8A03-5805-8402-6D10C6F9DA96

**Figure 8.**
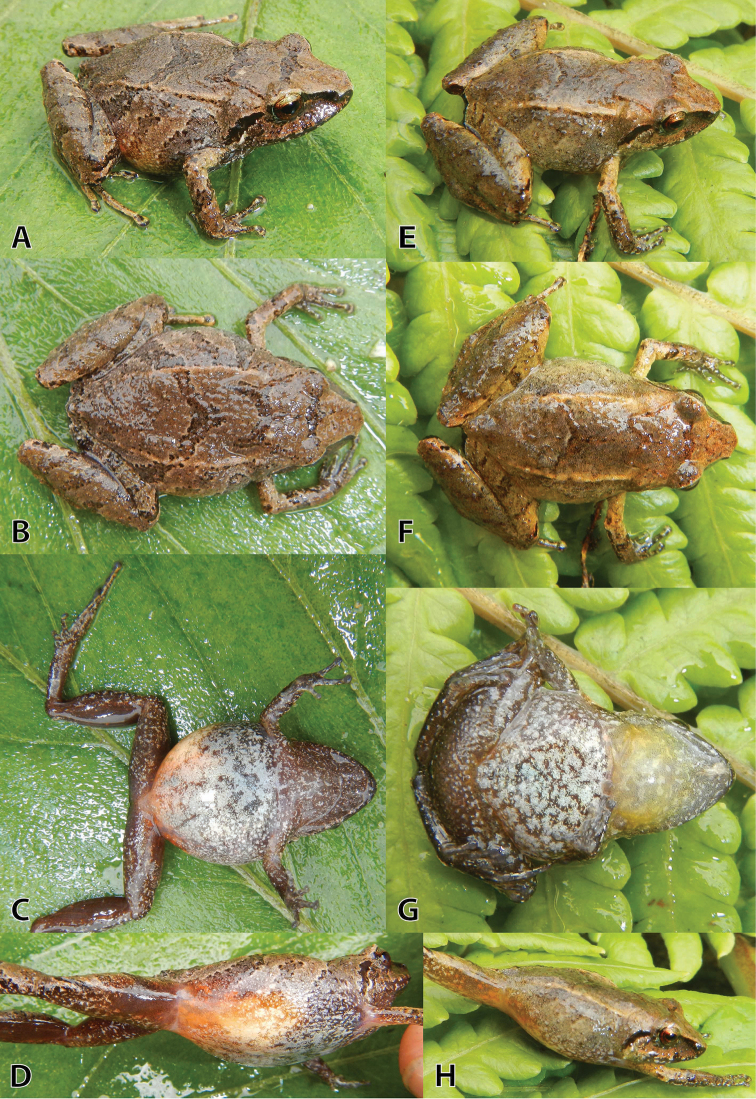
Life colouration of female (**A–D**, IWU 237, SVL 24.2 mm) and male (**E–H**, NMP-P6V 75067, SVL 15.5 mm) *Pristimantis albertus* in dorsolateral (**A, E**), dorsal (**B, F**), ventral **C, G**) and lateral (**D, H**) views. Photos by E. Lehr.

**Figure 9.**
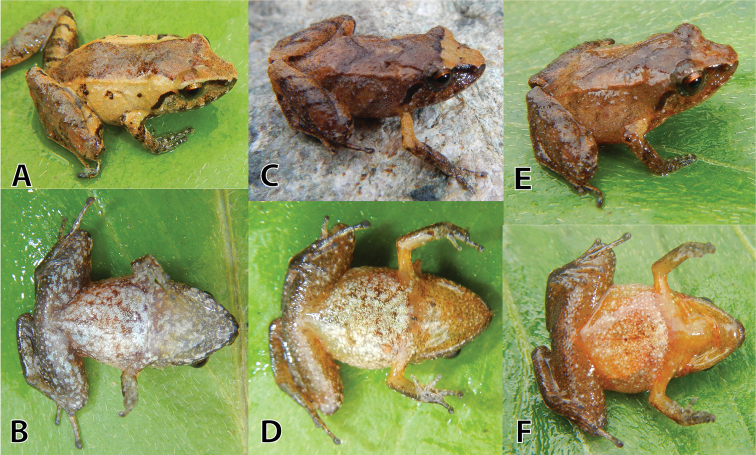
Colouration in life variation of males of *Pristimantis albertus***A, B** (MUSM 31956, SVL 13.7 mm) **C, D** (IWU 257, SVL 13.4 mm) **E, F** (MUSM 31959, SVL 12.9 mm). Photos by E. Lehr.

Figs 8 , 9 , Table 4

##### Material.

Five males: MUSM 31953, 31956, 31959; NMP-P6V 75067, 76021 (NMP-P6V 76021 GenBank accession numbers MW075394 (16S rRNA) and MW075415 (12S rRNA)), from the Pui Pui Protected Forest (Rio Huatziroki valley, 11°07'37.2"S, 75°10'37.0"W; Fig. 3), 1970 m a.s.l., Distrito Pichanaqui, Provincia Chanchamayo, Región Junín, Peru, collected on 13–16 June 2013 by Edgar Lehr, Jiří Moravec, Juan Carlos Cusi and Rudolf von May. Three females: MUSM 31957; NMP-P6V 75068, 76020 (NMP-P6V 76020 GenBank accession numbers MW075393 (16S rRNA) and MW075414 (12S rRNA), same locality and collecting data as the males.

##### Diagnostic characters.

In general, the newly-collected and genetically-determined individuals of *Pristimantis albertus* correspond to the description of the type specimens. However, they differ in the following features: (1) discoidal fold is present, weakly defined (discoidal fold absent, according to Duellman and Hedges 2007); (2) dentigerous processes of vomers are present (absent, according to Duellman and Hedges 2007); (3) groin is orange in both sexes (Figs 8C, D, H, 9D, F), the orange colouration can continue to posterior parts of flanks and anterior parts of thighs, rarely the orange colouration is present in the axils (MUSM 31959).

##### Measurements

**(Table 4).** The males are relatively small (SVL 12.9–19.5 mm, n = 5). All lack nuptial pads, but all have vocal slits, a distinct subgular vocal sack and, therefore, are regarded as adults. Females are represented by one adult (NMP-P6V 76020) and two subadult (MUSM 31957, NMP-P6V 75068) specimens. Snout-vent length of the adult female exceeds the size published for the type specimens (24.2 vs. 19.7–20.7 mm in Duellman and Hedges 2007).

**Table 4.** Measurements (in mm) of *Pristimantis albertus*. For abbreviations, see Material and methods.

Character	NMP-P6V 75067	NMP-P6V 76021	MUSM 1953	MUSM 31965	MUSM 31959	NMP-P6V 76020	NMP-P6V 75068	MUSM 31957
SEX	M	M	M	M	M	F	F	F
SVL	15.5	13.4	19.5	13.7	12.9	24.2	14.3	14.6
TL	8.6	7.3	9.0	7.6	7.0	12.3	7.8	8.0
FL	6.5	5.6	8.1	6.2	5.6	10.2	6.0	6.1
HL	6.1	6.0	7.7	6.1	5.5	9.6	5.7	6.3
HW	5.9	5.3	7.4	5.5	5.1	9.1	5.4	5.6
ED	2.1	1.7	2.2	1.7	1.8	2.5	1.9	1.8
TY	0.7	0.6	0.9	0.7	0.6	1.2	0.6	0.6
IOD	1.9	2.1	2.3	1.8	1.8	2.8	1.8	2.0
EW	1.2	1.2	1.7	1.3	1.0	1.6	1.0	1.1
IND	1.9	1.8	2.3	1.9	1.7	2.9	1.9	1.8

##### Colouration of males in life

**(Fig. 9).** The dorsal colouration resembles the colouration of the adult females. In one individual (MUSM 31956), the light brown colouration of the dorsum is sharply contrasting with the light tan flanks. The throat is brown with cream flecks, the belly is cream with brown mottling and the vocal sack is greenish-yellow.

##### Distribution and natural history.

Our record of *Pristimantis albertus* from the PPPF lies ca. 65 km straight SE of the species type locality and represents the first record of this species for the Region Junín (Fig. 3).

#### Pristimantis sagittulus

Taxon classificationAnimaliaAnuraStrabomantidae

(Lehr, Aguilar & Duellman, 2004)

BD5644EB-A8AA-5A90-99C5-EF6F3755AD42

##### Material.

One male: MUSM 31952, from the Pui Pui Protected Forest (Rio Huatziroki valley, 11°07'37.2"S, 75°10'37.0"W; Fig. 3), 1970 m a.s.l., Distrito Pichanaqui, Provincia Chanchamayo, Región Junín, Peru, collected on 14 June 2013 by Edgar Lehr, Jiří Moravec, Juan Carlos Cusi and Rudolf von May.

##### Distribution and natural history.

Our record of *Pristimantis sagittulus* from the PPPF lies ca. 65 km straight SE of the species type locality (Lehr et al. 2004) and represents the first record of this species for the Region Junín (Fig. 3).

### Comparative specimens examined

*Pristimantis aniptopalmatus* (1): Peru, Junín, buffer zone of the Pui Pui Protected Forest, 1970 m a.s.l., MUSM 33996, NMP-P6V 75055.

*Pristimantis ashaninka* (5): Peru, Junín: border of the Pui Pui Protected Forest, 1700 m a.s.l., MUSM 36517 (holotype), MUSM 32736, 32742, NMP-P6V 75063, 75064, all paratypes.

*Pristimantis bipunctatus* (1): Peru, Junín, buffer zone of the Pui Pui Protected Forest, 1970 m a.s.l., MUSM 31954.

*Pristimantis lindae* (3): Peru, Cusco: Alto Shima, 1785 m a.s.l., MUSM 26528, 26542; Alto Shima, 1790 m a.s.l., MUSM 26540.

## Discussion

With the description of *Pristimantis sinschi*, the number of species in this genus known from Peru rises to 140 (AmphibiaWeb 2020), 13 of which (*P. albertus*, *P. aniptopalmatus*, *P. ashaninka*, *P. attenboroughi*, *P. bipunctatus*, *P. bounides*, P. cf. platydactylus, P. cf. stictogaster, *P. cruciocularis*, *P. humboldti*, *P. puipui*, *P. sagittulus* and *P. sinschi*) have been recorded inside the PPPF or its close surroundings. Comparison with the anuran list of the more intensively surveyed Yanachaga-Chemillén National Park (YCNP; see Angulo et al. 2016), which is located ca. 100 km NW of the PPPF, reveals that seven (54%) *Pristimantis* species occurring in PPPF are unknown from the YCNP. This leads us to the conclusion that the anuran fauna of the isolated PPPF area is characterised by an unusually high degree of local endemism and, therefore, deserves adequate protection. We predict that the amphibian and reptile species richness of the PPPF is much higher than is currently known because large parts remain unexplored.

The description of *Pristimantis albertus* was based on two adult females obtained by Hedges in 1987 from Rio San Alberto, 2.1 km E of Oxapampa (Provincia Pasco, Peru) at 1970 m a.s.l. (Duellman and Hedges 2007). It was assigned to the *Pristimantis danae* species Group (Hedges et al. 2008) and briefly accounted for by Duellman and Lehr (2009). Later, it was reported and documented with colour photographs from the Yanachaga-Chemillén National Park (Angulo et al. 2016). Samples of three newly-collected specimens of *P. albertus* were included in genetic analyses performed by Lehr and von May (2017) and Lehr et al. (2017b). Nevertheless, no additional data on the distribution or morphology of this species were published. Our series of eight specimens of *P. albertus* collected in the PPPF includes the first males of the species and consequently leads us to update the diagnostic and phenotypical characters of the species.

With a long, acuminate snout and a broad, longitudinal, red stripe on the posterior surfaces of the thighs, *Pristimantis sagittulus* can easily be distinguished from all its congeners. Previously, the species was only known from its type locality at San Alberto, the Yanachaga-Chemillén National Park and adjacent localities at elevations of 1970–2479 m a.s.l. in the Region Pasco (Duellman and Lehr 2009; Angulo et al. 2016).

## Supplementary Material

XML Treatment for Pristimantis ventrimarmoratus

XML Treatment for Pristimantis sinschi

XML Treatment for Pristimantis albertus

XML Treatment for Pristimantis sagittulus
